# Invasive mold infection of the gastrointestinal tract: A case series of 22 immunocompromised patients from a single academic center

**DOI:** 10.1093/mmy/myac007

**Published:** 2022-01-29

**Authors:** Orlando Quintero, Libby Allard, Dora Ho

**Affiliations:** Division of Infectious Diseases and Geographic Medicine, Department of Medicine, 300 Pasteur Drive, Stanford, CA 94305-5107, USA; Department of Pathology, Stanford University School of Medicine, 300 Pasteur Drive, Stanford, CA 94305-5107, USA; Division of Infectious Diseases and Geographic Medicine, Department of Medicine, 300 Pasteur Drive, Stanford, CA 94305-5107, USA

**Keywords:** Gastrointestinal, immunocompromised, fungal infection, mold, transplant

## Abstract

Invasive mold infection (IMI) of the gastrointestinal (GI) tract is a rare complication in immunocompromised patients that carries a high mortality rate. It is most often described in the setting of disseminated disease. Early diagnosis and treatment are critical in its management, but this is rarely obtained, leading to delayed therapy. To describe the clinical characteristics, treatment and outcomes of this infection, we reviewed all the cases of adult patients with histopathological findings from autopsy or surgical specimens that demonstrated fungal invasion into the GI tract at Stanford Hospital & Clinics from January 1997 to August 2020. Twenty-two patients that met criteria were identified and they were all immunocompromised, either due to their underlying medical conditions or the treatments that they received. The most common underlying disease was hematological malignancies (63.6%) and the most common symptoms were abdominal pain, GI bleeding and diarrhea. A majority of patients (72.7%) had disseminated invasive mold infection, while the rest had isolated GI tract involvement. In 2/3 of our cases, the fungal genus or species was confirmed based on culture or PCR results. Given the very high mortality associated with GI mold infection, this diagnosis should be considered when evaluating immunocompromised patients with concerning GI signs and symptoms. A timely recognition of the infection, prompt initiation of appropriate antifungal therapy as well as surgical intervention if feasible, are key to improve survival from this devastating infection.

**Lay summary:**

Patients with a weakened immune system can suffer from mold infections in the bowel, which are difficult to diagnose and have very high death rate. We examined such cases in our institution in order to learn about their clinical and microbiological features. This study can further improve our understanding of these infections in order to improve patient outcome.

## Introduction

Invasive mold infection (IMI) is a life-threatening opportunistic infection that usually affects immunocompromised patients,^1,2^ particularly those with prolonged neutropenia and those receiving high-dose corticosteroids or immunosuppressive therapy.^2,3^ The incidence of IMI has been increasing during the last 20 years, as a consequence of the use of more effective but also more toxic chemotherapy and immunosuppressing regimens, in addition to the growing number of patients undergoing hematopoietic stem cell transplantation (HSCT) or solid organ transplantation (SOT).^2,4^ IMI most commonly involves the respiratory tract, including the lungs or sinuses but other organ systems may also be infected as a result of hematogenous spread.^1^ Gastrointestinal (GI) IMI, which is associated with high mortality, is a rarely seen form of extra-pulmonary fungal infection and is most often described in the setting of disseminated disease.^5^ Due to the lack of specific clinical signs and symptoms, early diagnosis is rarely obtained, leading to a high mortality rate. In fact, GI IMI is often diagnosed post-mortem, and in many cases, as a ‘surprising’ finding. To further facilitate our understanding of this rare, but frequently fatal opportunistic infection, we conducted a retrospective study to identify the clinical and microbiological features of GI IMI cases in our institution.

## Methods

This is a retrospective study of adult patients with histopathological findings from autopsy or surgical pathology specimens that demonstrated fungal invasion into the GI tract at Stanford Hospital & Clinics (SHC) from January 1997 to August 2020. Cases thought consistent with yeast colonization or infection (which might represent Candida or non-Candida species) were excluded. Twenty-eight patients that met criteria were identified through review of SHC's Pathology Department data base. Six patients were excluded due to incomplete records, resulting in 22 patients included in this cohort. Data was collected from patient's index hospitalization, which is defined as the hospitalization during which a GI IMI diagnosis was made antemortem or postmortem. The date of GI IMI diagnosis is defined as either the date when the GI tract specimen that demonstrated IMI was obtained by surgery or endoscopy, or the date of death with subsequent autopsy that confirmed GI IMI. IMI were classified as proven, probable or possible according to the definition from the European Organization for Research and Treatment of Cancer and the Mycoses Study Group Education and Research Consortium.^6^ All 22 GI IMI cases in this study met the criteria of proven IMI (i.e., histopathologic examination of a specimen obtained by biopsy in which hyphae are seen accompanied by evidence of associated tissue damage).^6^ Each patient's demographic, clinical and radiologic information as well as relevant pathology and microbiology results were extracted from Stanford's Research Repository and electronic medical record system (STARR; STAnford medicine Research data Repository), a clinical data warehouse containing live Epic data from SHC, the Stanford Children's Hospital, the University Healthcare Alliance and Packard Children's Health Alliance clinics and other auxiliary data from hospital applications such as radiology Picture Archiving and Communication System (PACS). Collected data were stored and managed using Redcap electronic data capture tools hosted at Stanford University. This study was approved by the Stanford Healthcare Institutional Review Board.

Fungal speciation of the GI IMI was achieved either by culture or by polymerase chain reaction (PCR)-sequencing. DNA was extracted from formalin-fixed, paraffin-embedded tissue and subjected to real-time PCR targeting ITS2 and D2 regions of fungal ribosomal RNA locus. Cycle sequencing was performed on PCR products, and the identity of sequences was determined using a public database.^7,8^

For statistical analysis, categorical variables are presented in counts and percentages; age was reported as median with interquartile range (IQR).

## Results

### Patient characteristics

All 22 patients of this cohort were notably immunocompromised, either due to their underlying medical conditions, or the treatments that they received. Demographic information and type of immunodeficiency are summarized in Table 1. The majority of the patients were male 13/22 (59%) and the median age was 52.5 (IQR = 26).

**Table 1.** Demographic information.

Patients with invasive fungal infection of the GI tract	22
Age, years, median (interquartile range)	52.5 (26)
Female, sex, n (%)	9 (40.9%)
Race, n (%)	
Asian	2 (8.6%)
Hispanic	4 (17.3%)
White	11 (50%)
Unknown	5 (21.7%)
Forms of immunodeficiency, n (%)	
Hematologic malignancy	14 (63.6%)
AML	5
ALL	4
HLH	2
MDS	1
CML	1
CLL	1
With hematopoietic stem cell transplant	9 (40.9%)
Allogeneic, matched related	1
Allogeneic, matched unrelated	6
Umbilical cord blood	2
Solid organ transplant	3 (13.6%)
Double lung	1
Heart-lung	1
Liver	1
Other	5 (22.7%)
Autoimmune hepatitis	2
HIV/AIDS	1
COPD	1
IABP and ECMO	1

**Abbreviations**: AIDS, acquired immune deficiency syndrome; ALL, acute lymphocytic leukemia; AML, acute myeloid leukemia; CLL, chronic lymphocytic leukemia; CML, chronic myeloid leukemia; COPD, chronic obstructive pulmonary disease; ECMO, extracorporeal membrane oxygenation; HIV, human immunodeficiency virus; HLH, hemophagocytic lymphohistiocytosis; IABP, intra-aortic balloon pump; MDS, myelodysplastic syndrome.

Fourteen of these patients (63.6%) suffered from hematologic malignancies; the majority had acute myeloid or lymphocytic leukemia, while the remaining had hemophagocytic lymphohistiocytosis (HLH), myelodysplastic syndrome or chronic myeloid leukemia or chronic lymphocytic leukemia (Table 1). Of those, 9 patients (64.2%) had undergone HSCT and were at a median of 102 days post-transplant. All were receiving immunosuppressive agents for either prophylaxis for or treatment of graft versus host disease (GVHD). Two of these HSCT patients (22.2%) failed to achieve engraftment at the time of death. For the other five patients with hematologic malignancies but without HSCT, GI IMI was diagnosed at a median of 149 days after the cancer diagnosis, and all were undergoing active chemotherapy. Three patients had SOT (13.6%) and were at a median of 162 days post-transplant when GI IMI was diagnosed. None of them had history of acute rejection and were all on standard immunosuppressive regimen per SOT protocols. Two patients (9%) had autoimmune hepatitis; both received steroids and one was additionally on azathioprine and tacrolimus. Other disease conditions in this cohort include chronic obstructive pulmonary disease (COPD; treated with chronic inhaled and systemic steroids), advanced human immunodeficiency virus/acquired immunodeficiency syndrome (HIV/AIDS; CD4 cell count of 29 cells/microliter prior to index hospitalization) and critical illness after a motor vehicle accident [requiring placement of an intra-aortic balloon pump (IABP) followed by extracorporeal membrane oxygenation (ECMO)].

At the time of the index hospitalization, a majority of these patients (20/22, 90.9%) were on active immunosuppressive therapy. Various agents were used but steroids and tacrolimus were most common, in 13 (59%) and 11 (47.8%) patients, respectively (Table 2).

**Table 2.** Detailed characteristics, treatment, microbiologic/pathologic findings and outcome of patients with gastrointestinal invasive mold infection.

							Antifungal therapy					
Case #	Age/sex	Underlying condition	Major immunosuppression	Neutropenia?*	Suspicions of IFI?	Other know infections within same admission**	Prophylaxis	Treatment	GI IMI diagnoses by:	Method of IMI diagnosis	Outcome	Final IMI diagnosis
1	61 F	UCB-HSCT For B-ALL	Failed engraftment	Yes	Probable aspergillosis, lungs	VRE bacteremia, HMPV PNA, HHV-6 viremia	Fluconazole → Posaconazole → Caspofungin	Isavuconazole + caspofungin	Autopsy	Antemortem (+) galactomannan; Postmortem culture (+) *A. fumigatus*	Death	Disseminated (lungs, GI - esophagus, stomach, colon)
2	24 M	MUD-HSCT for AML	Acute & chronic skin and GI GVHD	No	No	VRE bacteremia	Posaconazole → Caspofungin	/	Autopsy	Postmortem histopathology (+) ‘Aspergillus like’ fungal hyphae	Death	Disseminated (lungs, GI - stomach)
3	39F	MUD-allo HSCT for AML	Skin GVHD	No	No	*Candida parapsilosis* candidemia	Fluconazole	Anidulafungin	Autopsy	Postmortem histopathology (+) ‘Aspergillus like’ fungal hyphae	Death	Disseminated (brain, lungs, GI - stomach, small bowel, abdominal/pelvic serosa)
4	53M	MUD-allo HSCT for CML	Skin and GI GVHD	No	Possible IFI, lungs	MSSA bacteremia, parainfluenza PNA	Fluconazole	Caspofungin → Voriconazole	Autopsy	Postmortem culture (+) *A. fumigatus*	Death	Disseminated (heart, lungs, GI - stomach, kidneys)
5	56M	MUD-allo HSCT for AML	GI GVHD	No	Possible IFI, lungs, CNS	CoNS bacteremia	Fluconazole	Voriconazole + Ampho B (Ambisome)	Autopsy	Postmortem histopathology (+) ‘Aspergillus like’ fungal hyphae	Death	Disseminated (brain, thyroid, heart, lungs, GI - liver, small bowel, colon, rectum, kidneys)
6	65 M	MUD-allo HSCT for DLBCL	Skin and GI GVHD	No	Possible IFI, lungs, CNS	Influenza B PNA	/	Ampho B (Ambisome)	Autopsy	Postmortem culture (+) *A. fumigatus*	Death	Disseminated (brain, heart, lungs, GI – stomach, kidneys)
7	28 F	MUD-allo HSCT for MDS	GI, liver, eyes GVHD	No	Possible IFI, lungs	No	/	Ampho B (Ambisome)	Autopsy	Postmortem histopathology (+) ‘Aspergillus like’ fungal hyphae	Death	GI - stomach
8	62 M	AML	Consolidationchemotherapy	Yes	Possible IFI, lungs	No	/	Voriconazole	Autopsy	Postmortem histopathology (+) ‘Aspergillus like’ fungal hyphae	Death	GI - stomach
9	22 F	ALL	Clinical trial with mAb 216 + vincristine	Yes	Possible IFI, lungs	Clostridium septicum & MSSA bacteremia	/	Voriconazole →Anidulafungin	Autopsy	Postmortem histopathology (+) ‘Aspergillus like’ fungal hyphae	Death	Disseminated (Lungs, GI - small bowel, cecum)
10	60 M	ALL	Induction chemotherapy	Yes	Possible IFI, lungs	No	/	Voriconazole	Autopsy	Postmortem culture (+) Zygomycetes	Death	Disseminated (lungs, spleen, GI - stomach, liver)
11	62 M	HLH	Steroids, etopoxide	Yes	Possible IFI, lungs	No	/	Voriconazole → Caspofungin	Autopsy	Postmortem culture (+) *Rhizopus spp.*	Death	Disseminated (lungs, GI - small and large bowels)
12	18 M	HLH	Anakinra, rituximab, steroids, etoposide	No	No	No	Caspofungin	/	Autopsy	Postmortem culture (+) *Rhizopus spp*.	Death	Disseminated (lungs, GI - stomach, liver, mesenteric artery, portal vein, spleen)
13	58 F	Double lung transplant	Mycophenolate, tacrolimus	No	Probable IFI, lungs	No	Itraconazole	Caspofungin → Ampho B (Ambisome)	Autopsy	Antemortem respiratory culture and postmortem lung culture (+) *Rhizopus spp.*	Death	Disseminated (lungs, GI - large bowel)
14	54 F	Liver transplant	Tacrolimus, steroids	Yes	Proven aspergillosis (peritonitis)	Hepatitis E, *E coli* bacteremia, *C. difficile* colitis, CMV viremia	Voriconazole → anidulafungin	/	Autopsy	Postmortem culture (+) *A. niger*	Death	Disseminated (brain, heart, lungs, GI- stomach and large bowel, kidneys)
15	45 F	heart-lung transplant	ATG, tacrolimus, steroids, mycophenolate	No	Probable aspergillosis (lungs)	No	Caspofungin	Isavuconazole	Autopsy	Postmortem culture (+) *A. fumigatus*	Death	Disseminated (lungs, trachea, mediastinum, GI - esophagus)
16	24 F	Autoimmune hepatitis	Steroids	No	No	MSSA bacteremia	/	Caspofungin	Autopsy	Postmortem culture (+) *A. fumigatus*	Death	Disseminated (lungs, GI - stomach)
17	52 F	Autoimmune hepatitis	Azathioprine, steroids, tacrolimus	No	No	MSSA bacteremia	/	/	Autopsy	Postmortem culture (+) *Scedosporium apiosperm*	Death	Disseminated (brain, heart, lungs, GI -stomach, kidneys)
18	41 M	MRD-allo HSCT for ALL	Steroids, tacrolimus	No	No	No	Fluconazole → voriconazole	Voriconazole + caspofungin → Ampho B (Ambisome) + posaconazole + caspofungin	Surgical pathology from colectomy and nephrectomy	Histopathology (+) fungal hyphae, confirmed as *Rhizopus spp.* by PCR	Alive	Disseminated (GI – Ileum, transverse colon, left kidney)
19	34 M	UCB-HSCT for AML	Mycophenolate, tacrolimus	Yes	No	VRE bacteremia, *Streptococcus mitis* bacteremia, *Burkholderia multivorans* bacteremia, HHV-6 viremia, adenovirus pneumonitis, parainfluenza pneumonitis, BK viruria	Isavuconazole before admission for lung nodules then voriconazole from day 0 of hsct then caspofungin	caspofungin	Surgical pathology from small bowel resection	Histopathology (+) fungal hyphae, confirmed as *Rhizopus spp.* by PCR	Death	GI - omentum, small bowel
20	84 M	COPD	Steroids	No information	No	No	fluconazole	Ampho B (Abelcet)	Surgical pathology from sigmoid colectomy	Histopathology (+) ‘mucor like’ fungal hyphae	Death	GI - colostomy stoma, colon
21	41 M	AIDS	No	No	Proven otitis with Aspergillus fumigatus and probable aspergillosis of the sinuses and lungs	Disseminated *Mycobacterium avium complex*	/	anidulafungin	Surgical pathology from EGD	Histopathology (+) ‘Aspergillus like’ fungal hyphae	Alive	GI – Esophagus
22	56 M	IABP → ECMO	No	No	No	No	/	Amph B (Ambisome) + caspofungin	Surgical pathology from partial gastrectomy	Histopathology (+) fungal hyphae, confirmed as *Rhizopus spp.* by PCR	Death	GI – stomach

*Neutropenia is defined as an absolute neutrophil count number <1000 cells/µl × >= 7 days on index admission.

**Other known infections within the same admission is defined as all infections excluding urinary tract infections or mucocutaneous viral infections.

**Abbreviations:** AIDS, Acquired immunodeficiency syndrome; ALL, Acute lymphocytic leukemia; Allo, Allogeneic; AML, Acute myeloid leukemia; Ampho B, Amphotericin B; ATG, anti-thymocyte globulin; B-ALL, B-cell acute lymphoblastic leukemia; CML, Chronic Myeloid Leukemia; CML; Chronic Myeloid Leukemia, CMV, Cytomegalovirus; COPD, Chronic obstructive pulmonary disease; CNS, central nervous system; CoNS; Coagulase-negative staphylococci; DLBCL, Diffuse large B-cell lymphoma; ECMO: extracorporeal membrane oxygenation; F, female; GI, Gastronintestinal; GVHD, graft versus host disease; HHV-6, Human Herpesvirus 6; HLH, Hemophagocytic lymphohistiocytosis; HMPV, Human metapneumovirus; HSCT, hematopoietic stem cell transplantation; IABP, intra-aortic ballon pump; IFI, invasive fungal infection; IMI, Invasive mold infection; M, Male; MDS, Myelodysplastic syndromes; MSSA, Methicillin-sensitive Staphylococcus aureus; MRD, matched related donor; MUD, Matched unrelated donor; PCR, polymerase chain reaction; PNA, pneumonia; UCB, unrelated cord blood; VRE, Vancomycin-resistant enterococci.

### Signs and symptoms of GI IMI

Most frequent GI symptoms were abdominal pain (n = 10; 45.4%), diarrhea (n = 8; 36.3%), GI bleeding (n = 6; 27.2%) as well as nausea and vomiting (n = 6; 27.2%). The most common physical examination findings included abdominal tenderness (n = 10; 45.4%), abdominal distension (n = 9; 40.9%) and fever (n = 6; 27.2%). Two patients (9%) did not have any documented GI symptoms and 3 patients (13.6%) reportedly had a normal GI physical exam (Table 3).

**Table 3.** Signs and symptoms of patients with gastrointestinal invasive mold infection.

Signs and symptoms of GI IMI	Number (n); (%)
**Most Frequent GI symptoms**	
Abdominal pain	10/22; (45.4%)
Diarrhea	8/22; (36.3%)
GI bleeding	6/22; (27.2%)
Vomiting and nausea	6/22; (27.2%)
Low appetite	4/22; (18.1%)
No GI symptoms	2/22; (9%)
**Physical examination findings prior to GI IMI diagnosis**	
Abdominal tenderness	10/22; (45.4%)
Abdominal distension	9/22; (40.9%)
Fever	6/22; (27.2%)
Jaundice	3/22; (13.6%)
Decreased bowel movements	3/22; (13.6%)
Hypothermia	1/22; (4.5%)
Normal physical exam	3/22; (13.6%)

**Abbreviations:** GI, gastrointestinal; IMI, invasive mold infection.

Fifteen patients (68.1%) underwent computerized tomography (CT) scan of the abdomen. All had abnormal but variable findings, including colitis (as demonstrated as bowel wall thickening; n = 6; 40%), pneumatosis (n = 4; 26.6%), bowel distention (n = 3, 20%) and increased fat stranding of the colon with ascites (n = 3; 20%) (Table 4).

**Table 4.** CT and endoscopic findings of patients with gastrointestinal invasive mold infection.

CT and endoscopic findings	Number (n); (%)
**Major CT abdomen findings**	
Wall thickening small and large bowel	6/15; (33%)
Pneumatosis	4/15; (26.6%)
Increased fat stranding of the colon and ascites	3/15; (20%)
Distention of multiple bowel loops	3/15; (20%)
Infarction or intramural hemorrhage	2/15; (13.3%)
Ileus or bowel obstruction	2/15; (13.3%)
**Endo-colonoscopy findings**	
Erythematous mucosa of upper GI tract	3/12; (25%)
Erythematous mucosa of lower GI tract	2/12; (16.6%)
Ulceration of upper GI tract	2/12; (16.6%)
Ulceration of lower GI tract	4/12; (33.3%)
Severe colitis	1/12; (8.3%)
**Endoscopic biopsy results**	
GVHD of stomach	5/6; (83.3%)
GVHD of colon	4/6; (66.6%)
GVHD of duodenum	4/6; (66.6%)
Duodenum with extensive crypt loss and focal crypt apoptosis	1/6; (16.6%)
Histopathology positive fungal hyphae	1/6; (16.6%)

**Abbreviations:** CT, Computed tomography; GVHD, graft versus host disease.

Nine patients (40.9%) underwent upper and/or lower endoscopy of the GI tract. A total of 7 esophagogastroduodenoscopies (EGD) and 5 colonoscopies were performed. The most common endoscopic findings were erythematous mucosa or ulceration of the GI tract. Biopsy of the GI mucosa was performed in 6 pts and revealed GVHD in 83.3% and fungal elements were found in only one specimen. (Table 4).

### Characteristics of the fungal infections and antifungal therapy

Among all 22 patients, GI IMI was suspected in only one patient antemortem. However, a significant number of patients had suspected or proven IMI diagnosis outside the GI tract (n = 14). Proven IMI included aspergillus peritonitis (n = 1) and aspergillus otitis media (n = 1). Four patients had probable IMI in lungs (n = 4) and one of these patients also had sinus involvement. Eight patients had possible IMI of the lungs (n = 8) and with additional central nervous system involvement in two patients (Table 2).

Twelve patients, including seven with HSCT and three with SOT, were on antifungal prophylaxis during or prior to the index hospitalization. The other two patients had HLH and COPD, respectively. The majority received either an echinocandin (n = 6) or fluconazole (n = 4). Prophylactic regimen was changed in some patients due to transaminitis (e.g., with posaconazole changed to caspofungin) or when ‘mold-coverage’ was deemed necessary (e.g., with fluconazole changed to voriconazole). For eight of these patients, prophylaxis was subsequently changed to treatment when IMI was suspected or demonstrated (Table 2).

Nine patients without prior antifungal prophylaxis were started on antifungal therapy during the index hospitalization as treatment of IMI or as empiric therapy for sepsis or neutropenic fever. Antifungal agents employed included an echinocandin (n = 6), amphotericin B (lipid formulation; n = 4), voriconazole (n = 3) as well as isavuconazole (n = 1), or combinations of the above. One patient (4.5%) did not receive any antifungal agents.

**Figure 1.**
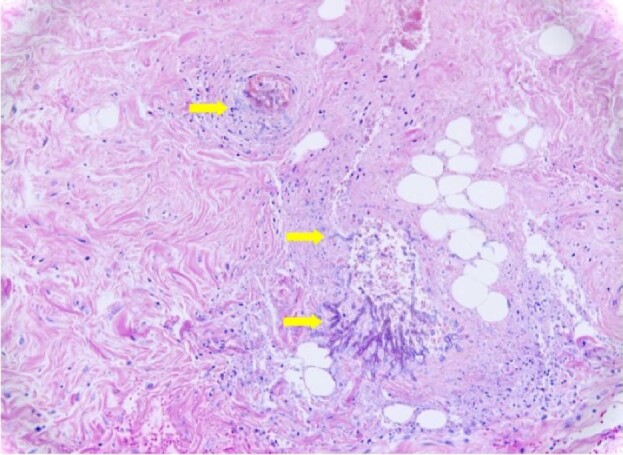
Histopathologic findings from autopsy of Case #5 (see Table 2 for details). Hematoxylin and eosin stain, 20× colon tissue with angioinvasive fungal hyphae (yellow arrows) with accompanying necrosis and minimal tissue reaction.

**Figure 2.**
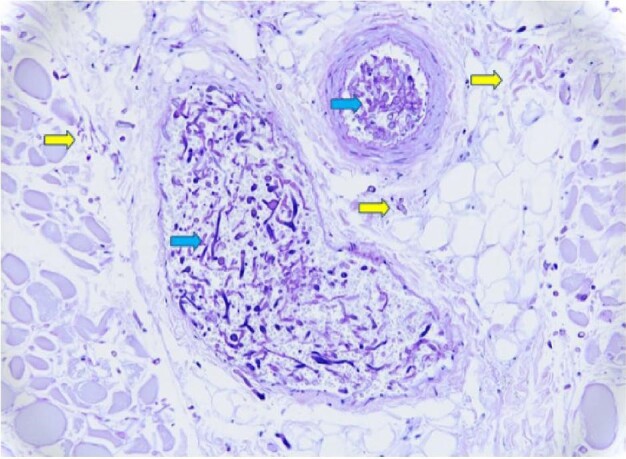
Histopathologic findings from autopsy of Case #11 (see Table 2 for details). Periodic Acid-Schiff Stain with diastase (PAS-D) stain of colon (original magnification 20×) shows Rhizopus hyphae within blood vessels (blue arrows) and associated scattered invasive forms (yellow arrows). No cellular tissue reaction is present.

GI IMI were confirmed in all 22 cases by histopathological findings on autopsy or surgical pathology samples, with invasion of fungal hyphae into the GI tract mucosa. Histopathologic findings of GI IMI are illustrated in Figures 1 and 2. Fungal speciation was available in 14 cases by culture of the specimens (n = 11; 78.5%) or by PCR-sequencing of the paraffin embedded tissues (n = 3; 21.4%). Identified fungal species included *Aspergillus spp*. (n = 6), Mucorales (n = 7) and *Scedosporium apiospermum* (n = 1).

Per autopsy findings, the stomach was the most common site of GI tract involvement (n = 12), followed by large bowel (n = 6), small bowel (n = 4), liver (n = 3) and esophagus (n = 2). Sixteen patients (72.7%) had disseminated infection, while 6 (27.2%) had IMI involving the GI tract alone. For those cases with dissemination, all of them (n = 16, 100%) had lung IMI; 5 patients (31.2%) also had CNS involvement. For the surgical pathology cases, either upper (n = 3) or lower (n = 2) GI tract was involved, as detailed in Table 2.

## Discussion

IMI of the GI tract is an infrequent disease that usually affects immunocompromised host, representing less than 5% of the total of IMI in this population.^9,10^ In our cohort, the patients were also notably immunocompromised, either due to their underlying medical conditions, or the treatments that they received. In recent years, the incidence of GI IMI has increased likely due to environmental and host factors, higher index of suspicion, as well as improvement in diagnostic techniques.^10^ However, despite widespread access to noninvasive diagnostics and invasive procedures for biopsy, timely diagnosis of GI IMI remains challenging due to its non-specific clinical presentation. Previous case series or reviews on intestinal aspergillosis or mucormycosis have mostly relied on collection of cases from the literature^5,9,11^ or from multiple medical centers.^2^ To our knowledge, our case series represents the largest cohort for GI IMI from a single academic center.

In our study, the most common underlying disease was hematological malignancies, including 64% of these patients who had also undergone HSCT. Due to their underlying malignancy and associated treatment, these patients not only had increased risks for opportunistic infections, but they were also prone to acquire other infectious and non-infectious pathologies of the GI tract that can mimic and/or further increase risks for GI IMI. Notoriously, the signs and symptoms as well as radiographic findings of GI IMI are very non-specific. In this cohort, the most common symptoms of abdominal pain, GI bleeding and diarrhea, or CT findings of bowel wall thickening or pneumatosis could be seen with many other GI diseases in these patient populations, including neutropenic enterocolitis and GI GVHD. In fact, only one patient of this cohort was suspected to have GI IMI during the index hospitalization.

About 3/4 of our patients had disseminated IMI, while 1/4 had isolated GI tract involvement. For disseminated disease, it likely starts with primary pulmonary infection with subsequent hematogenous spread to other organs including the GI tract.^1^ For isolated GI IMI, focal invasion after ingestion of food (or even medications) contaminated with fungal spores would be the most likely scenario.^2,9^ A recent review found that GI aspergillosis mostly affects the lower GI tract, with 61% of cases confined to the small bowel and 21% to the large intestine.^11^ Another study reported that upper GI IMI was more common in SOT patients, while lower GI infection was more frequent in hematologic/HSCT patients.^5^ However, in our cohort, the upper GI tract was the predominant site of involvement (n = 12, 54.5%). Isolated lower GI tract involvement was found in six patients (27.2%) and four patients (18.1%) had concomitant upper and lower tract IMI. This trend of upper GI tract predominance persists even when only patients with hematologic malignancies (with or without HSCT) or only those with aspergillosis were included in the analysis.

As described in previous studies,^2,5,12^ prolonged use of systemic steroids is a major risk factor for IMI. Steroids were also the most common immunosuppressants in our cohort, used in 59% of the patients, followed by tacrolimus in 47.8%. For the nine patients with HSCT, six had documented GVHD, with five affecting the GI tract. While patients with GVHD would require enhanced immunosuppression in general, GI tract involvement might further increase the risk of GI IMI. For instance, the immune-mediated destruction of the intestine mucosa can increase risk of fungal invasion and the immune dysregulation from GVHD would further cripple the immune response to the fungal infection.

About half of the patients were given antifungal prophylaxis, mostly with fluconazole or an echinocandin. While fluconazole is notably inadequate as prophylaxis against mold infection, four of the six patients on echinocandin prophylaxis developed IMI with *Aspergillus spp.*, or ‘Aspergillus-like’ organisms as demonstrated by histopathology. Echinocandins have activities against *Aspergillus spp.*, but certainly, breakthrough aspergillosis with echinocandins has been well documented.^13^ The other two patients on either itraconazole or voriconazole as prophylaxis developed GI IMI with *Rhizopus spp*., consistent with the lack of activities against *Mucorales* with these two azoles.

One major strength of our study is the confirmation of the fungal genus or species in 2/3 of the cases. The most common fungal species found in autopsy were *Aspergillus spp.* and *Rhizopus spp*., based on culture or PCR results. For previous case series,^5,9^ many of the cases were diagnosed via histopathologic examination of biopsy specimens only, and cultures were either not obtained or did not yield any growth of fungal organisms. However, fungal speciation based on morphology features in histologic and/or cytologic can be incorrect in >20% of cases.^14^ In fact, one of our cases was caused by *Scedosporium apiospermum* and its histopathologic findings in tissue specimens would be indistinguishable from those with *Aspergillus spp.*

For diagnostics, endoscopy is a common modality used for the evaluation of the GI tract. The decision to perform EGD versus colonoscopy (or both) likely based on individual patient's presenting signs and symptoms. For HSCT patients, such procedures were frequently employed to rule out GVHD of the GI tract. Six patients underwent endoscopy, but only one patient received an IMI diagnosis based on EGD despite gross endoscopic abnormalities (e.g., erythematous mucosa or ulceration) noted in ∼90% of the cases. For the other five patients with endoscopy, biopsy specimens from the GI tract did not reveal any fungal elements. Such low diagnostic yield by endoscopy might be due to sampling error or difficulties in differentiating gut mucosal changes from concomitant processes (e.g., GVHD vs GI IMI) by endoscopic examination alone. Thus, even a negative endoscopic biopsy of abnormal mucosa does not necessarily exclude IMI. This observation further underscores the difficulty in making this diagnosis antemortem.

In a retrospective series of GI aspergillosis,^2^ galactomannan (GM) antigenemia testing was performed in 20 cases, with a positive result in 16. Thus, a positive GM in the presence of GI signs or symptoms can potentially alarm the possibility of GI aspergillosis. Similar testing for fungal antigens is not available for mucormycosis, but PCR-based testing, such as detection of plasma cell-free fungal DNA fungal by PCR, has been found increasing useful and can play major roles in fungal diagnosis for aspergillosis, mucormycosis and other fungal infections.^15,16^

Mortality rate of IMI in general depends on several factors, including patient population studied, fungal species involved, specific organ(s) infected as well as timing of initiating appropriate treatment. For instance, with HSCT as the underlying disease condition, fungal involvement of the central nervous system or infection with highly resistant fungi likely confers the worst outcome.^17,18^ From prior reviews, mortality rate of GI mucormycosis^5^ was estimated at 57%, while that of GI aspergillosis was 39%.^11^ In our study, all but one patient died during the index hospitalization. Although patients in this cohort had many comorbidities, such high mortality rate may be attributed to missed or delayed diagnosis of IMI, as nine patients in our cohort were not suspected to have IMI at all, and in some cases with GI mucormycosis, the antifungal-agent(s) employed did not have activities against Mucorales. In their systemic review of GI aspergillosis,^11^ Yelika *et al.* reported that 63% of patients treated with surgery survived, compared with 46% treated with antifungal therapy alone. Thus, timely diagnosis and initiation of appropriate antimicrobial therapy as well as surgical intervention when applicable, are crucial to improve patient outcome.

Our study has several limitations. Given the retrospective nature of this study, we relied on chart review to determine patients’ physical examination findings and symptoms associated with their GI IMI, but such documentation may not be accurate. Further, since a majority of our patients had GI IMI confirmed by autopsy findings, patients that survived the infection would be missed. Thus, the findings of our study likely reflect those of the sickest patients but may not be applicable to patients with less severe disease. Finally, this study was conducted at a single center; results may not be wholly generalizable to centers with different immunosuppression protocols, antifungal prophylaxis strategies and/or diagnostic methods, which may vary substantially.

In conclusion, given the very high mortality associated with GI IMI, a timely recognition of the infection is crucial to improve outcome. The clinical and radiographic findings of GI IMI are nonspecific. Thus, it is prudent to consider this diagnosis when evaluating immunocompromised patients with concerning GI symptoms or findings, and in particular, if they fail to respond to therapy for the more common GI pathologies such as GHVD in HSCT. Early diagnosis with prompt initiation of appropriate antifungal therapy as well as surgical intervention are key to improve survival from this devastating infection. While current diagnostic modalities to detect GI IMI are very limited, newer technologies such as detection of cell-free fungal DNA in plasma by PCR^15,16^ may provide a rapid and non-invasive diagnostic venue when surgical biopsy of the GI tract is not feasible. However, the sensitivity and specificity of such technology for GI IMI are yet to be defined.
